# Supplementary material for: PIM1 Inhibition Affects Glioblastoma Stem Cell Behavior and Kills Glioblastoma Stem-like Cells
Source: Int J Mol Sci. 2021 Oct 15;22(20):11126. doi: 10.3390/ijms222011126 (PMC8541331; doi:10.3390/ijms222011126)
Supplement: Supplementary file 1 [file ijms-22-11126-s001.zip › ijms-1405222-supplementary.pdf]

Supplementary Information to the manuscript entitled

PIM1 inhibition affects the glioblastoma stem cell behavior and kills glioblastoma stem-like cells

Carolin Seifert[1,2], Ellen Balz[1,2], Susann Herzog[1], Anna Korolev[1], Sebastian Gaßmann[1], Heiko Paland[1,2], Matthias A. Fink[1,2], Markus Grube[1], Sascha Marx[2], Gabriele Jedlitschky[1], Mladen V. Tzvetkov[1], Bernhard H. Rauch[1], Henry W. S. Schroeder[2], Sandra Bien-Möller[1,2]

[1] Institute of Pharmacology, University Medicine Greifswald

[2] Department of Neurosurgery, University Medicine Greifswald

Corresponding Author: Sandra Bien-Möller, PhD
Institute of Pharmacology/C_DAT
Department of Neurosurgery
University Medicine Greifswald
Felix-Hausdorff-Str. 3
17487 Greifswald, Germany
Phone: +49 3834 865646
FAX: +49 3834 865631
Email: sandra.bien@med.uni-greifswald.de

S1 Antibodies used for Western Blot Analyses

The following primary antibodies were diluted in TBST and 0.5 % BSA and incubated overnight at 4°C: monoclonal mouse anti-CD44 (Cell Signaling Technology, Boston, USA), mouse anti-CD133 (Merck Millipore, Darmstadt, Germany; Novus Biologicals, Cambridge, UK), monoclonal mouse anti-Nestin (Acris Antibodies GmbH, Herford, Germany; StemCell Technologies Germany GmbH, Cologne Germany; BioLegend, Inc., San Diego, CA, USA) as well as polyclonal goat anti-Nestin (Santa Cruz Biotechnology, Inc., Dallas, Texas, USA), monoclonal rabbit anti-PIM1 (for PIM1S, Epitomics, Burlingame, USA; abcam®, Cambridge, UK; Cell Signaling Technology, Boston, USA), monoclonal rabbit anti-PIM1 (for PIM1L, LSBio, LifeSpan Technologies, Inc, Seattle, USA; Cell Signaling Technology, Boston, USA), polyclonal goat anti-PIM1 (Santa Cruz Biotechnology, Inc., Dallas, Texas, USA), and monoclonal mouse anti-GAPDH (Meridian Life Science, INC., Memphis, USA). The secondary horseradish peroxidase-conjugated goat-anti-mouse and goat-anti-rabbit IgG antibodies (both Bio-Rad Laboratories GmbH, Munich, Germany) and horse-anti-goat (Vector Laboratories, Burlingame, CA) were used at a 1:1000 dilution for 1 to 1.5 hours at room temperature. Detection of chemiluminescence signals was done with the *ChemiDoc™ XRS Imaging System* (Bio-Rad, Hempstead, U.K.) using *ECL Plus Western Blotting Substrate* (Thermo Scientific, Rockford, USA) followed by densitometric analysis (Quantity One, Bio-Rad). The relative optical densities of the specific bands were measured and normalized to GAPDH as loading control.

S2 Immunofluorescence staining

The cells were fixed a second time in ethanol containing 5 % acetic acid for 10 minutes at -20 °C followed by three washing steps with PBS. The sectioned cells were blocked in PBS containing 0.3 % Triton X-100 and 5 % mouse serum (PAN™ Biotech GmbH, Aidenbach, Germany), 5 % rabbit serum (PAN™-Biotech GmbH, Aidenbach, Germany) or 5 % goat serum (GIBCO® by life technologies™, Carlsbad, California, USA.; Thermo Fisher Scientific, Waltham, Massachusetts, USA). The following primary antibodies were diluted in PBS containing 1 % BSA and 0.3 % Triton X-100 and incubated at 4 °C overnight: monoclonal mouse anti-CD44, 1:50 (Cell Signaling Technology, Boston, USA); mouse anti-CD133, 1:100 (Merck Millipore, Darmstadt, Germany; Novus Biologicals, Cambridge, UK), monoclonal mouse anti-Nestin, 1:50 (Acris Antibodies GmbH, Herford, Germany; BioLegend, Inc., San Diego, CA, USA) as well as polyclonal goat anti-Nestin, 1:30 (Santa Cruz Biotechnology, Inc., Dallas, Texas, USA), monoclonal rabbit anti-PIM1, 1:50 (Cell Signaling Technology, Boston, USA) and polyclonal rabbit anti-PIM1, 1:50 (LS Bio, LifeSpan Technologies, Inc, Seattle, USA) (both PIM1 antibodies were mixed half and half) as well as polyclonal goat anti-PIM1, 1:30 (Santa Cruz Biotechnology, Inc., Dallas, Texas, USA). Negative controls were treated with PBS containing 1 % BSA and 0.3 % Triton X-100 without any primary antibody. On the next day, the sections were washed three times with PBS and the secondary antibodies were incubated for 2.5 hours at room temperature in a 1:150 dilution: chicken anti-goat Alexa Fluor® 488 (life technologies™

and invitrogen MOLECULAR PROBES®, Carlsbad, USA; Thermo Fisher Scientific, Waltham, Massachusetts, USA), donkey anti-goat Alexa Fluor® 568, donkey anti-rabbit Alexa Fluor® 568 (both life technologies™, Foster City, USA; Thermo Fisher Scientific, Waltham, Massachusetts, USA), chicken anti-rabbit Alexa Fluor® 488 (invitrogen MOLECULAR PROBES®, Carlsbad, USA; Thermo Fisher Scientific, Waltham, Massachusetts, USA), goat anti-rabbit Alexa Fluor® 568, goat anti-mouse Alexa Fluor® 488, chicken anti-mouse Alexa Fluor® 488 nm (all from life technologies™ and invitrogen MOLECULAR PROBES®, Carlsbad, USA; Thermo Fisher Scientific, Waltham, Massachusetts, USA). Afterward the sections were washed with PBS three times before embedding in Dako Fluorescence Mounting Medium (Carl Roth GmbH & Co. KG, Karlsruhe, Germany). The sections were dried flat overnight before being examined at the Zeiss Axio Observer D1 (Carl Zeiss, Jena, Germany).

S2 Caspase 3 assay

Pelleted cells were rinsed with PBS two times and centrifuged again at 250 x g for 10 minutes. The supernatant was gently removed and discarded while the cell pellet was lysed by the addition of 60 µl Lysis Buffer (provided in the kit) and incubated on ice for 10 minutes followed by centrifugation at 10,000 x g for 1 minute. 50 µl of cell lysate and 50 µl of 2X Reaction Buffer 3 were mixed and given in a well of 96-well-plate. Prior to using the 2X Reaction Buffer 3, 10 µl of DTT stock (provided by the kit) per 1 ml of 2X Reaction Buffer 3 were added. To each reaction well, 5 µl of Caspase-3 substrate (DEVD-AFC, 1 mM) were added and the plate was incubated at 37 °C for 1 to 2 hours as suggested by the supplier. Then the plates were read on a microplate reader (Infinite M200 Microplate reader, TECAN, Crailsheim, Germany) using an excitation wavelenght of 400 nm and an emission wavelenght of 505 nm.

Supplemental Figure S4

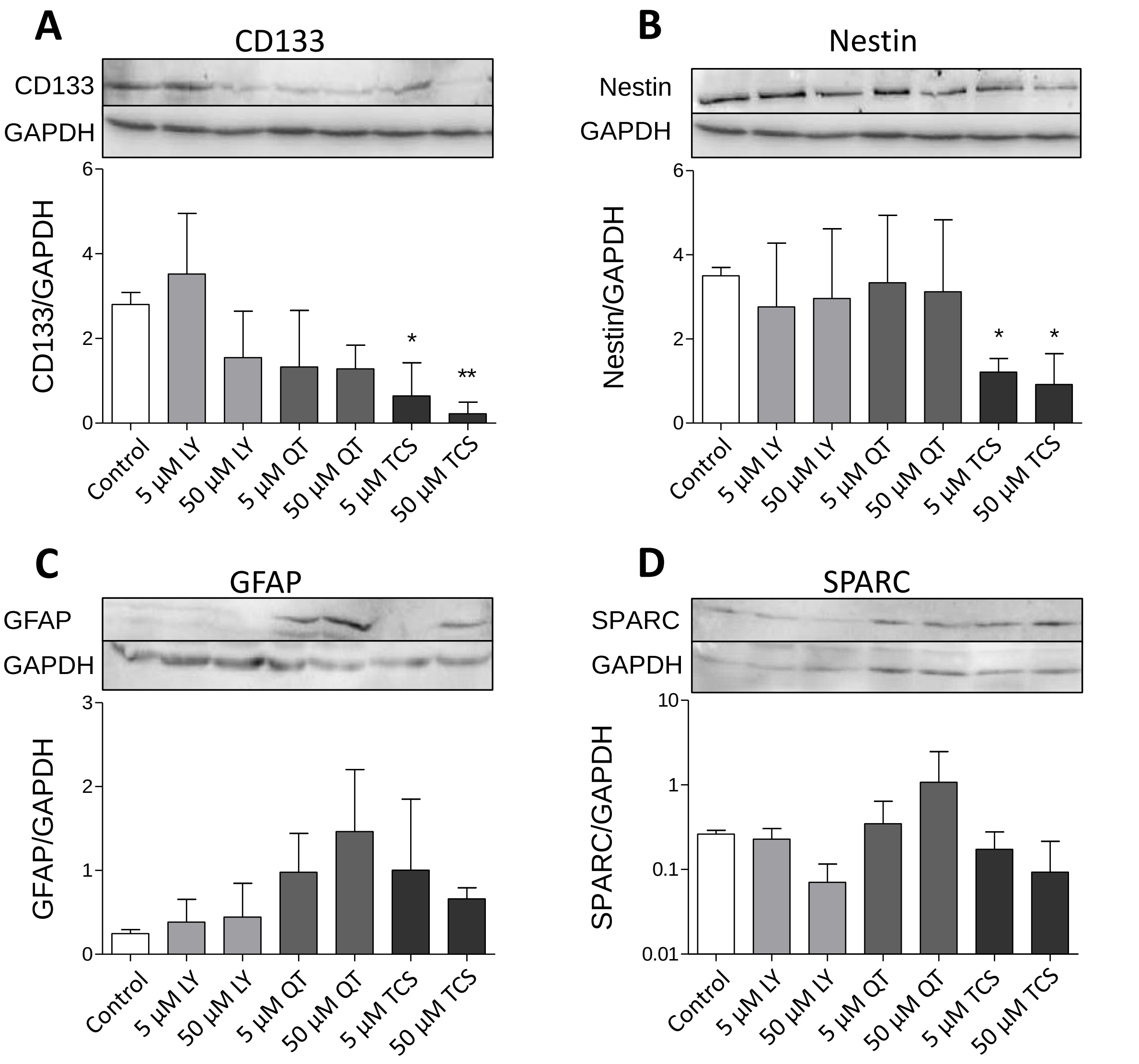

Supplemental Figure S4. Pharmacological PIM1 blocking regulates expression of stemness and differentiation marker proteins in the murine GBM cell line GL261. Cells were treated each with 5 or 50 µM of the dual PI3K/PIM1 inhibitor LY294002 (LY), of the selec-tive PIM1 inhibitors quercetagetin (QT) or of TCS PIM1-1 (TCS). The respective solvent, dimethylsulfoxide (DMSO), was added to the control cells. (**A-D**) Protein expression of CD133 (**A**), Nestin (**B**), GFAP (**C**) and SPARC (**D**) in GL261 cell lysates determined by immunoblotting using specific antibodies. GAPDH served as loading control. Results are representative of three to four independent experiments. Columns represent means and SD, OneWay ANOVA with Dunnetts Multiple Comparison Test, significant differences for $^*p<0.05$ and $^{**}p<0.005$
